# Enhanced sparrow search algorithm with DV-Hop for high-precision fire sensor localization in underground spaces

**DOI:** 10.1371/journal.pone.0338706

**Published:** 2025-12-17

**Authors:** Jiang Li, Liliang Dong, Le Xu, Fangqiong Luo, Zhenkun Lu, Yajian Huang, Shenghan Wei

**Affiliations:** 1 School of Electrical Engineering, Hunan Polytechnic of Water Resources and Electric Power, Changsha, Hunan, China; 2 School of Mathematics and Computer Engineering, Guangxi Science & Technology Normal University, Laibin, Guangxi, China; 3 School of Computer Science and Technology, Guizhou University, Guiyang, Guizhou, China; 4 School of Electronic Information, Guangxi Minzu University, Nanning, Guangxi, China; University of Southampton, MALAYSIA

## Abstract

Fire monitoring in underground spaces is critical for emergency response, yet traditional localization methods like DV-Hop suffer from significant localization errors due to hop count ambiguity and premature convergence in optimization. To address these issues, we propose an Enhanced Sparrow Search Algorithm with Improved DV-Hop (ESSADV-Hop) method. The method incorporates a golden ratio-based communication radius division strategy to refine hop count granularity and an enhanced sparrow search algorithm with Gaussian perturbations to escape local optima. Experimental results show that ESSADV-Hop reduces the average localization error by 55.7% compared to DV-Hop (from 0.2910 to 0.1288) and outperforms other variants by 11.74%∼23.05% in accuracy, demonstrating its effectiveness for fire sensor localization in complex underground environments.

## 1 Introduction

Underground spaces in cities, such as subway stations, underground shopping centers, and parking lots, pose significant fire risks [1]. When a fire occurs, smoke spreads rapidly due to the complex structure of these buildings, making rescue operations more challenging [2]. Therefore, it is critical to choose the most appropriate technical approach for fire localization in underground spaces. Existing fire localization techniques primarily consist of range-based localization algorithms and range-free localization algorithms [3].

The range-based localization algorithms mainly include received signal strength indicator (RSSI) [4], time of arrival (TOA) [5], time difference of arrival (TDOA) [6], and angle of arrival (AOA) [7]. In addition, RSSI, TOA, and TDOA algorithms suffer from relatively large localization errors in complex environments due to signal propagation may be affected by other factors. In addition, the signal propagation of the AOA is difficult to control exactly in complex environments. At the same time, the localization error is defined as the normalized euclidean distance between estimated and actual node positions, which is normalized by communication radius.

The range-free localization algorithms mainly include center of gravity algorithm [8], convex programming algorithm [9], and DV-Hop algorithm [10]. Among these, DV-Hop is widely adopted due to its low power consumption and strong adaptability to heterogeneous environments [11]. At present, the improvement of DV-Hop is divided into two types. Improve the hop count or hop distance of DV-Hop to enhance the localization accuracy. For example, Li et al. [12] proposed a novel DV-Hop algorithm based on dual communication radius and used it for sensor node localization. Kaushik et al. [13] proposed a 3-D DV-Hop algorithm based on the information of nearby nodes and used it for sensor node localization. Cao et al. [14] proposed an improved DV-Hop localization algorithm based on nodes negotiation and multi communication radius. Mohanta et al. [15] proposed an orthogonal learning CTO-based DV-Hop localization algorithm for three-dimensional wireless sensor networks. Shi et al. [16] proposed an improved DV-Hop algorithm. The localization accuracy is improved by the swarm intelligence algorithm. Cui et al. [17] proposed a localization algorithm based on improved DV-Hop and DE. Zhou et al. [18] introduced bacterial foraging optimization to the DV-Hop, which achieved significantly higher positioning accuracy than the basic DV-Hop. OuYang et al. [19] proposed an improved adaptive genetic algorithm to handle the aforementioned problem and used a modified evaluation function to reduce the error of distance measurement in a topological structure. Peng et al. [20] proposed an adaptive chaotic slime mold algorithm to optimize node localization in wireless sensor networks. Fute et al. [21] proposed FPSOTS and RSSI methods to evaluate inter-sensor distances. Yu et al. [22] proposed a gray wolf localization algorithm based on beetle antennae search.

Although the DV-Hop has been successfully applied in many fields, this method also suffers from the problem of low localization accuracy. According to the characteristics of node localization optimization problem for fire monitoring, we propose an improved DV-Hop algorithm (IDV-Hop) based on enhanced sparrow search algorithm (ESSADV-Hop). The method mainly includes three key points.

1) For fire sensor nodes in complex underground spaces, the DV-Hop roughly marks the hop count between the nodes as one hop when the nodes are within the communication radius, which results in a low localization accuracy of the nodes. Therefore, we use a golden ratio method to divide the communication radius of the fire sensor nodes to refine the hop counts between the nodes.

2) In order to further enhance the localization accuracy of fire-sensor nodes, we introduce an enhanced sparrow search algorithm (ESSA). Specifically, ESSA incorporates Gaussian perturbations with adaptive position updates to evade local optima and thus reduce node localization errors.

3) Finally, we first adopt the IDV-Hop to make a preliminary estimation of the fire sensor node position. The fire sensor node localizations are then passed to the ESSA algorithm. Then, the ESSA algorithm is used to further optimize the fire sensor node localization and calculate the position error. In addition, existing hybrid methods rely on fixed communication radius, failing to adaptively refine hop count resolution in complex underground space. On the contrary, ESSADV-Hop subdivides the communication radius and implements the correction of the hop count, which can effectively reduce the localization error.

Section 2 introduces the DV-Hop and sparrow search algorithm. In section 3, we analyze and improve the algorithm. Section 4 compares and analyzes the performance of the algorithms. Section 5 summarizes this work.

## 2 Algorithm introduction

### 2.1 DV-Hop algorithm

The traditional DV-Hop can be divided into three stages: determining the minimum hop count, calculating the average hop distance, and estimating the unknown node. The procedure for DV-Hop is as follows.

1) The beacon node broadcasts the data table containing its unique ID, position information, and initial hop count of 0 to other nodes in the communication range by flooding, and each node only saves the minimum hop count between other nodes around it.

2) After multiple floods, the anchor node obtains the minimum hop count and position information between the anchor node and other surrounding anchor nodes, and calculates the average hop distance *Hop*_*m*_ according to Eq (1).

$${Hop}_{m}=\frac{\sum_{m\neq n}\sqrt{{\left(x_{m}-x_{n}\right)}^{2}+{\left(y_{m}-y_{n}\right)}^{2}}}{\sum_{m\neq n}h_{mn}}$$ (1)

where $\left(x_{m},y_{m}\right)$ and $\left(x_{n},y_{n}\right)$ denote the positions of the anchor nodes *m* and *n*, respectively. The *h*_*mn*_ denotes the minimum hop count between anchor nodes *m* and *n*. The *Hop*_*m*_ is the average hop distance of anchor node *m*. Then, the estimated distance *d*_*um*_ between the unknown node and the anchor node can be expressed as in Eq (2).

$$d_{um}=Hop_{m}\cdot h_{um}$$ (2)

where *d*_*um*_ denotes the estimated distance from the unknown node *u* to the anchor node *m*. The *h*_*um*_ represents the minimum hop count from the unknown node *u* to the anchor node *m*.

3) The position of unknown node is calculated by the least square method.

### 2.2 Sparrow search algorithm

The sparrow search algorithm (SSA) is derived from the foraging and anti-predation behaviors of sparrow, which is composed of discoverers, followers, and watchers [23,24]. The discoverer is responsible for finding foraging areas and directions. The follower then uses the discoverer’s information to obtain food, and the watcher makes a safe judgment about the area where the sparrow population is located. Due to its faster convergence rate and higher solution accuracy, SSA has been applied to node localization optimization problems for fire monitoring to improve node localization accuracy.

Assuming that there exists a *D*-dimensional search space with *N* sparrows. The position of sparrow *i* at the *t*-th iteration represents a candidate solution $X_{id}^{\;1.5ptt}$ (i=1,2,...,N, t=1,2,...,T, d=1,2,...,D), where *T* is the maximum number of iterations. In the SSA algorithm, the discoverer can guide the sparrow population to search for food sources according to Eq (3).

$$X_{id}^{\;1.5ptt+1}=\left\{ \begin{array}{c} X_{id}^{\;1.5ptt}\cdot exp\left(-\frac{i}{\alpha \cdot T}\right),R_{2}<ST\\ X_{id}^{\;1.5ptt}+Q\cdot L,R_{2}\geq ST \end{array}\right.$$ (3)

where $X_{id}^{\;1.5ptt+1}$ is the new position of crow *i* at the (t+1)-th iteration. The *α* and *Q* denote a random number. The *L* indicates a matrix of 1×d in which every element is 1, $R_{[}2]\in [0,1]$ is warning value, and ST∈[0.5,1] is the safe value. When *R*_2_<*ST*, it indicates that the feeding area of the sparrow is safe, and the current discover can expand the range to search for food. When $R_{2}\geq ST$, it indicates that the sparrow’s feeding area is not safe and they need quickly fly to a safe area.

Next, the follower obtains food from the information of the discoverer, and the position update formula is as follows Eq (4).

$$X_{id}^{\;1.5ptt+1}=\left\{ \begin{array}{c} Q\cdot exp\left(\frac{X_{worst}^{\;1.5ptt}-X_{id}^{\;1.5ptt}}{i^{2}}\right),i>\frac{N}{2}\\ X_{best}^{\;1.5ptt+1}+\left|X_{id}^{\;1.5ptt}-X_{best}^{\;1.5ptt+1}\right|\cdot A^{+}\cdot L,otherwise \end{array}\right.$$ (4)

where $X_{worst}^{\;1.5ptt}$ is the worst position at the *t*–*th* iteration, $X_{best}^{\;1.5ptt+1}$ is the optimal position at the *t* + 1−*th* iteration, and the *A*^ + ^ represents a matrix of 1×d in which every element is 1 or -1. When $i>\frac{N}{2}$, it indicates that the each sparrow is very hungry and needs to fly quickly to an area with abundant food.

Finally, the watcher makes safe judgments about the area of the sparrow population described in Eq (5).

$$X_{id}^{\;1.5ptt+1}=\left\{ \begin{array}{c} X_{best}^{\;1.5ptt}+\beta \cdot |X_{id}^{\;1.5ptt}-X_{best}^{\;1.5ptt}|,f_{i}\neq f_{g}\\ X_{id}^{\;1.5ptt}+K\cdot \frac{\left|X_{id}^{\;1.5ptt}-X_{worst}^{\;1.5ptt}\right|}{f_{i}-f_{w}+\tau },f_{i}=f_{g} \end{array}\right.$$ (5)

where $X_{best}^{\;1.5ptt}$ is the optimal position at the *t*–*th* iteration. The *β* denotes the step size control parameter, K∈[−1,1] is a random number, and *f*_*i*_ represents a fitness value of sparrow *i*. The *f*_*g*_ and *f*_*w*_ are the current global optimal and worst fitness values, respectively. The *τ* is the smallest constant so as to avoid zero-division-error.

## 3 Analysis and improvement of algorithm

In this section, we analyze the error of the DV-Hop algorithm and propose an improved version called IDV-Hop. To further enhance the localization accuracy of fire nodes, we introduce the ESSA algorithm to optimize the IDV-Hop.

### 3.1 Error analysis of DV-Hop

**Fig 1:**
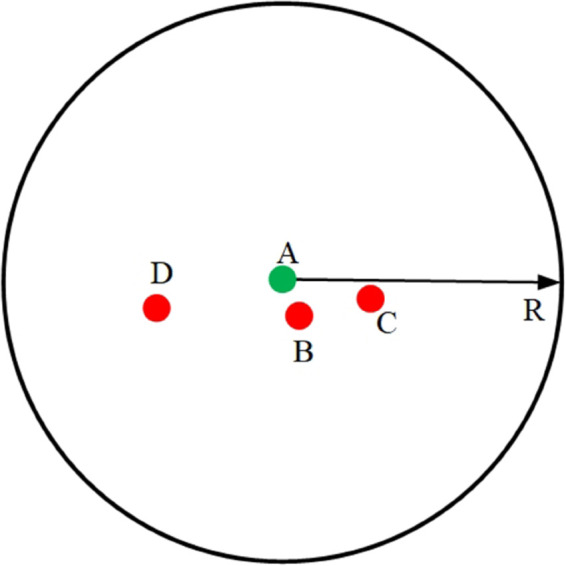
Hop count error analysis of DV-Hop.

In DV-Hop, all nodes within radius *R* are recorded as 1-hop regardless of actual distance (e.g., nodes B, C, D in Fig 1). This ignores distance variance, leading to inflated average hop-distance error. Our method resolves this via fractional hop counts.

As shown in Fig 1, assuming that the anchor node A communicates with nodes B, C, and D, the hop counts of node A with nodes B, C, and D are all 1. Moreover, the distances AB, AC, and AD differ so much that the actual distance between nodes with hop count 1 is not equal to the communication radius. Hence, this mechanism of hop count generates errors.

### 3.2 Hop count correction of DV-Hop

Based on the error analysis of the DV-Hop, it can be seen that roughly recording the number of hops as 1-hop affects the localization accuracy due to the large difference in the distance between nodes. Therefore, we use the strategy of symmetry and equal contraction of the golden ratio method to divide the communication radius, i.e., the communication radius is reduced by 0.618 times each time, and the golden division point is taken as the number of hops to reduce the localization error. The detailed steps of the golden ratio method are as follows:

Step 1: Define the golden ratio parameter. Let the golden ratio ϕ=0.618. The communication radius *R* is divided into *P* levels, where the division point for each level calculated based on the golden ratio *ϕ*.

Step 2: Calculate division points. For each level *P*, the division point *pos*_*p*_ is calculated as Eq (6).

$$pos_{p}=R\cdot \phi^{P-p}$$ (6)

where *P* represents the total division levels.

Step 3: Hop count record. According to the actual distance *dis* between nodes, the hop count *h* is calculated as Eq (7):

$$h=\left\{ \begin{array}{c} \phi^{P-1},0<dis\leq R\phi^{P-1}\\ \phi^{P-p},R\phi^{P-p+1}<dis\leq R\phi^{P-p} \end{array}\right.$$ (7)

where, *p*( 2≤p≤P)indicates the current level and *P* is the total division levels.

**Fig 2:**
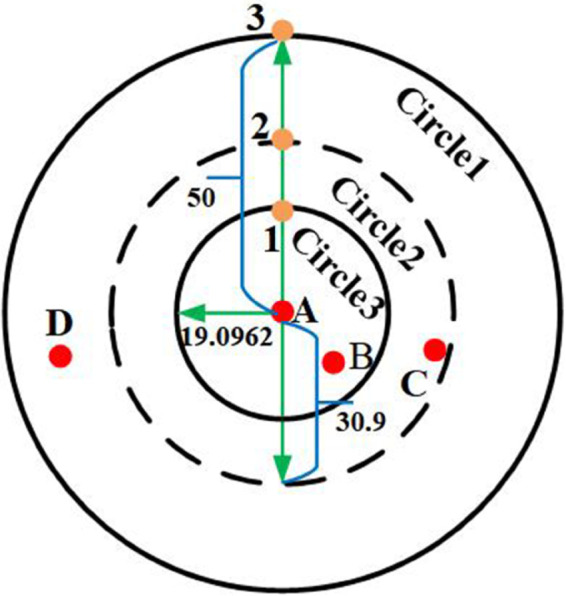
The golden ratio method divides the communication radius.

As shown in Fig 2, take *R* = 50, *P* = 3. We use the golden ratio method to divide the communication radius *R* into three times, marking the partitioned point *a*,*b* as the golden ratio point. The calculation of dividing point *d*_*i*_ and hop count *h* is as follows:

1) The calculation of dividing point. The $pos_{1}=50\cdot 0.618^{3-1}=19.0962$, $pos_{2}=50\cdot 0.618^{3-2}=30.9$, and $pos_{3}=50\cdot 0.618^{3-3}=50$.

2) Hop count record. If $0<dis_{AB}\leq 19.0962$, then $h_{AB}=0.618^{3-1}=0.382$. If $19.0962<dis_{AC}\leq 30.9$, then $h_{AC}=0.618^{3-2}=0.618$. If $30.9<dis_{AD}\leq 50$, then $h_{AD}=0.618^{3-3}=1$.

By dividing the communication radius by the golden ratio method, the hop count can be recorded more accurately, and the positioning error can be effectively reduced. Specifically, if two nodes are within the communication radius, DV-Hop records the hop count as 1, otherwise 0. In contrast, the golden ratio method is used to divide the communication radius into multiple levels (e.g., *P* = 3), with each level corresponding to a different hop count (e.g., 1, 0.618, and 0.382). The golden ratio ϕ=0.618 ensures symmetry and proportional scaling during radius division. Its mathematical property $\phi^{2}=1\;-\;\phi$ guarantees consistent geometric contraction, refining hop-count granularity while preserving topological relationships between nodes.

### 3.3 Improvement of SSA

In the node localization optimization for fire monitoring, the SSA is prone to miss the search for optimal fire sensor nodes and only search for fire sensor nodes within a small range, which makes the node localization accuracy low.

**Fig 3:**
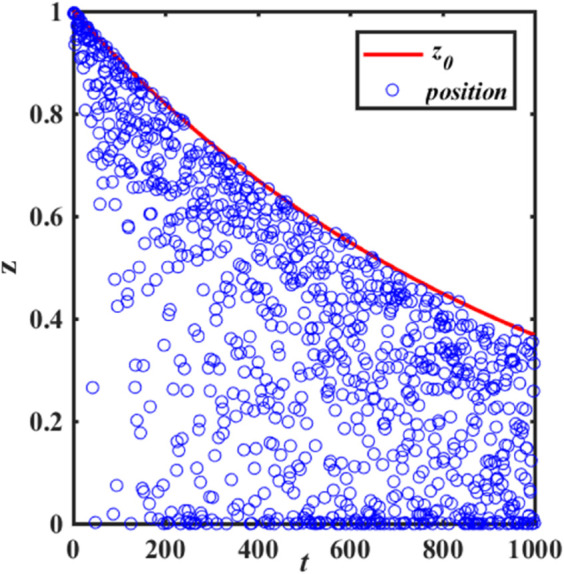
Trends in sparrow discover position before improvement.

**Fig 4:**
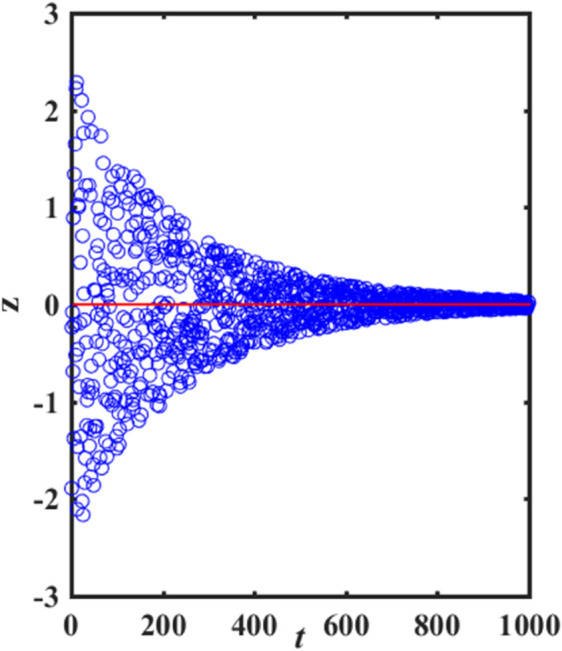
Trends in sparrow discover position after improvements.

It can be seen from Eq (3) that when *R*_2_<*ST*, the value of $z_{0}=exp(-t/\theta \;\cdot \;T)$ tends to 0 as the number of iterations increases. Fig 3 and Fig 4 show the change trend of sparrow discover position before and after improvement, respectively. As shown in Fig 3, the position of each sparrow is getting smaller after each iteration, and its convergence to the optimal solution is by moving close to the zero point. Thus, this strategy has strong local search capability around zero, but is prone to miss the optimal solution at non-zero and get stuck in local optima. As shown in Fig 4, the improvement strategy can update the position from both positive and negative directions of itself, which improves the search space of the sparrow algorithm, shown in Eq (8).

$$X_{id}^{\;1.5ptt+1}=\left\{ \begin{array}{c} X_{id}^{\;1.5ptt}\cdot tt,R_{2}<ST\\ X_{id}^{\;1.5ptt}+Q\cdot L,R_{2}\geq ST \end{array}\right.$$ (8)

tt=2·a·rand(1)−a (9)

a=0.5·π·acos(1−exp(−8·t)/T) (10)

where *tt* is calculated in Eq (9), and *a* in Eq (9) is described in Eq (10).

The follower of the sparrow will converge to 0 during the iteration and its position update pattern has a jump, which results in skipping the optimal solution. Therefore, we introduce a Gaussian perturbation strategy in the follower position update to improve the algorithm’s ability to find the optimal solution, describe in Eq (11).

$$X_{id}^{\;1.5ptt+1}=\left\{ \begin{array}{c} rand(1)\cdot Gaussian\cdot exp(\frac{X_{worst}^{\;1.5ptt}-X_{id}^{\;1.5ptt}}{i^{2}}),i>N/2\\ X_{best}^{\;1.5ptt+1}+|X_{id}^{\;1.5ptt}-X_{best}^{\;1.5ptt+1}|\cdot A^{+}\cdot L,otherwise \end{array}\right.$$ (11)

$$Gaussian=\frac{1}{\sigma \sqrt{2\pi }}\cdot exp(-\frac{(X_{id}^{\;1.5ptt}-\mu {)}^{2}}{2\sigma^{2}})$$ (12)

where μ=0 in Eq (12) is the mean value and $\sigma^{2}=0.5$ is the variance.

### 3.4 The execution flow of ESSADV-Hop

ESSADV-Hop is a node localization optimization method that combines IDV-Hop and ESSA. While hybrid methods combining DV-Hop with swarm intelligence algorithms (e.g., DE [17] and BFO [18]) have improved localization accuracy, they still face critical limitations:

1) Coarse hop count granularity due to fixed communication radius division, leading to persistent errors in distance estimation.

2) Premature convergence caused by inadequate exploration-exploitation balance in optimization algorithms.

The procedure is divided into the following steps. Firstly, the node parameters are initialized, and the communication radius is divided into multiple levels (e.g., *P* = 4) by using golden ratio, and the hop number between nodes is refined to reduce positioning error. Then, IDV-Hop is used for coarse localization to compute the initial distance between the unknown node and the beacon node, and the result is passed to ESSA. ESSA dynamically optimizes node localizations to avoid getting trapped in local optima through bidirectional search finder, Gaussian perturbation follower, and adaptive localization update strategy. In the iteration process, the sum of the differences between the actual distance and the estimated distance is used as the fitness function, and the position of the sparrow population is continuously updated until the maximum number of iterations is reached, and the optimal estimated position of the unknown node is finally output. Algorithm 1 is a pseudo-code for ESSADV-Hop.

**Algorithm 1. The pseudo-code of ESSADV-Hop.**



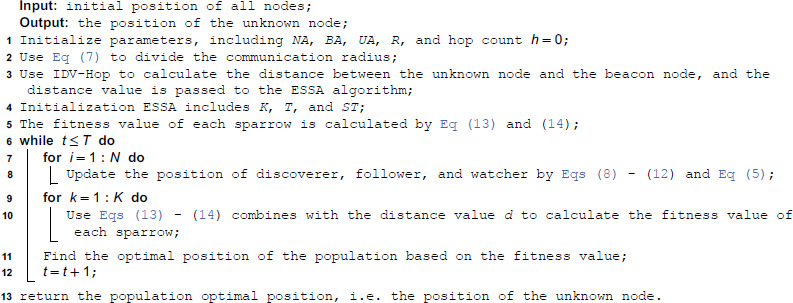



### 3.5 Time complexity of ESSADV-Hop

The time complexity is a crucial metric for evaluating the execution efficiency of algorithms. The computational complexity of evolutionary optimization algorithms refers to the number of operations involved in addition, subtraction, multiplication, and division during algorithm execution.

The time complexity of ESSADV-Hop consists of two components: IDV-Hop and ESSA. The gold ratio method in IDV-Hop refines hop counts with a complexity of O(P·n), while the least squares method has a time complexity of *O*(*n*^3^). For ESSA, the time complexity of population initialization is O(N·D). The time complexity of the ESSA after iteration is O(T·N·D). Thus, the total time complexity of ESSADV-Hop is $O(n^{3})\;+\;O(T\;\cdot \;N\;\cdot \;D)$. In addition, the total time complexity of DV-Hop is *O*(*n*^3^)). The GA and PSO methods have similar complexity with O(T·N·D). In summary, ESSADV-Hop has one more time complexity *O*(*n*^3^) than GA and PSO.

## 4 Comparison with different algorithms

In this section, we perform experiments on a PC configured with an Intel(R) Core(TM) i7-12700K CPU @ 3.61GHz and 32GB of RAM, and the programming software is Matlab R2020a. In Sec. 4.1, we compare IDV-Hop with DV-Hop. In Section 4.2, various algorithms are presented for experimental comparison. We assume that the size of the underground space is 100m * 100m. The algorithm uses Eq (13) as the objective function to calculate the fitness value, and uses Eq (14) to calculate the localization error of unknown nodes.

$$fitness=\sum_{u=1}^{N}\left|\sqrt{(x_{m}-x_{u}{)}^{2}+(y_{m}-y_{u}{)}^{2}}-d_{um}\right|$$ (13)

where *fitness* is the fitness value of fire sensor nodes. The (*x*,*y*) and $\left(x_{u},y_{u}\right)$ are the positions of anchor node and unknown node, respectively.

$$error=\frac{\sum_{u=1}^{N}\sqrt{(x_{u}-x_{u}^{\prime }{)}^{2}+(y_{u}-y_{u}^{\prime }{)}^{2}}}{UNA\times R}$$ (14)

where the $\left(x_{u},y_{u}\right)$ is the actual coordinates of the unknown node, the $\left(x_{u}^{\prime },y_{u}^{\prime }\right)$ is the estimated coordinates of the unknown node, *UNA* is the number of unknown node, *R* is the communication radius, and the *error* is the localization error. In simulations, actual coordinates $\left(x_{u},y_{u}\right)$ are predefined for validation. ESSA optimizes estimated positions $\left(x_{u}^{\prime },y_{u}^{\prime }\right)$ using distances *d*_*um*_ from IDV-Hop, without prior knowledge of $\left(x_{u},y_{u}\right)$.

### 4.1 Selection and validation of IDV-Hop algorithm

#### 4.1.1 Effect of communication radius on localization error.

**Fig 5:**
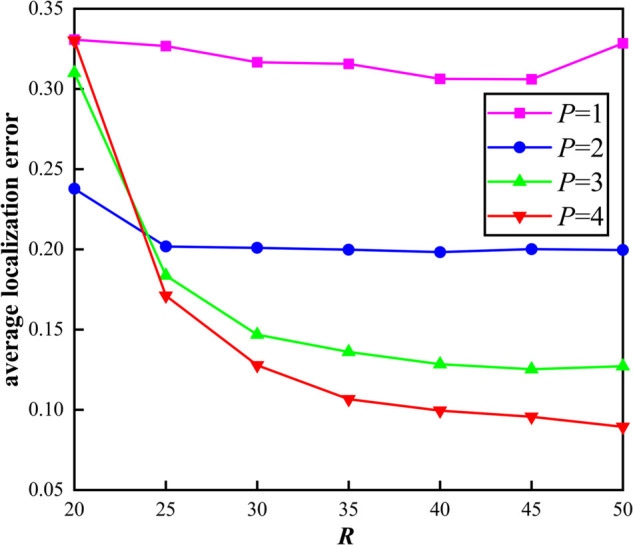
Localization error of different communication radius.

We assume that 100 fire sensor nodes are randomly deployed in underground spaces, including 30 anchor nodes, 70 unknown nodes, and the communication radius ranging from 20 to 50. The results are shown in Fig 5.

As can be seen from Fig 5, with the increase of the level *P*, the average localization error gradually decreases, which indicates that the more levels of communication radius division, the more accurate the hop count between fire sensor nodes and the lower the localization error. Under the same series (e.g. *P* = 4), the average localization error decreases with the increase of communication radius, indicating that the larger the communication radius, the more neighbor nodes of each fire sensor node, and the stronger the connectivity of the fire sensor network.

#### 4.1.2 Effect of number of anchor nodes on localization error.

**Fig 6:**
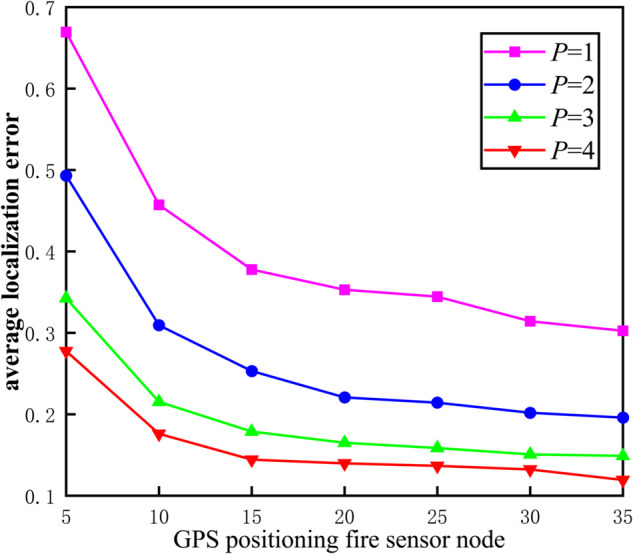
Localization error of different number of beacon nodes.

We assume that 100 fire sensor nodes are randomly deployed in underground spaces. The communication radius of all nodes is 30 and the range of anchor nodes is 5 to 35. The results are shown in Fig 6.

As shown in Fig 6, the average localization error gradually decreases as the increase of the level *P*, which indicating the subdivision of the communication radius can make the hop count between the fire sensor nodes more accurate. When *P* = 4, the average localization error decreases with the increase of the number of beacon nodes, indicating that the more the number of beacon nodes, the more accurate the algorithm estimates the localization of unknown fire sensor nodes.

To summary, the average localization error of the algorithm decreases with the increase of the level *P*, and the average localization error is the lowest when *P* = 4. Therefore, we choose the level *P* = 4 to divide the communication radius.

#### 4.1.3 Verification of IDV-Hop algorithm.

**Fig 7:**
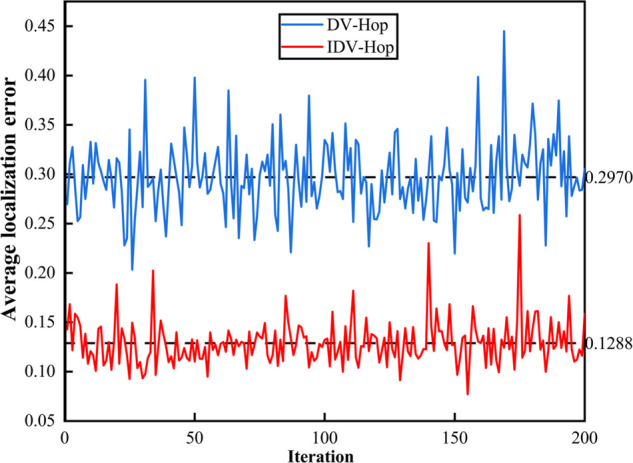
The performance of DV-Hop and IDV-Hop.

To verify the effectiveness of the IDV-Hop, this section introduces the DV-Hop for comparison, and the two algorithms are simulated 200 times, respectively. We assume that 100 fire sensor nodes are randomly deployed in underground spaces, the communication radius is 30 m, the beacon nodes are 30, and the unknown nodes are 70. The results are shown in Fig 7.

As shown in Fig 7, the average localization error of DV-Hop and IDV-Hop algorithms is 0.2970 and 0.1288, respectively, which is an improvement of 56.63 percent, indicating that IDV-Hop can effectively reduce the localization error by the golden ratio method. From the fluctuation point of view, the localization errors of both algorithms fluctuate to some extent. In contrast, IDV-Hop shows less fluctuations, indicating that its localization results are more stable and reliable. Therefore, IDV-Hop shows better performance than DV-Hop in terms of average localization error and stability, which validates the effectiveness of the golden ratio method.

### 4.2 Algorithm performance

In this section, we combine seagull optimization algorithm (SOA) [25], SSA, sand cat swarm optimization (SCSO) [26], and reptile search algorithm (RSA) [27] algorithms with DV-Hop to form SOADV-Hop, SSADV-Hop, SCSODV-Hop, and RSADV-Hop algorithms. In addition, we introduce genetic algorithm (GA) [28], particle swarm optimization (PSO) [29], goat optimization algorithm (GOA) [30], and polar lights optimizer (PLO) [31] methods to test. Finally, we compare these algorithms with DV-Hop, IDV-Hop and ESSADV-Hop.

#### 4.2.1 Effect of communication radius on localization error.

**Fig 8:**
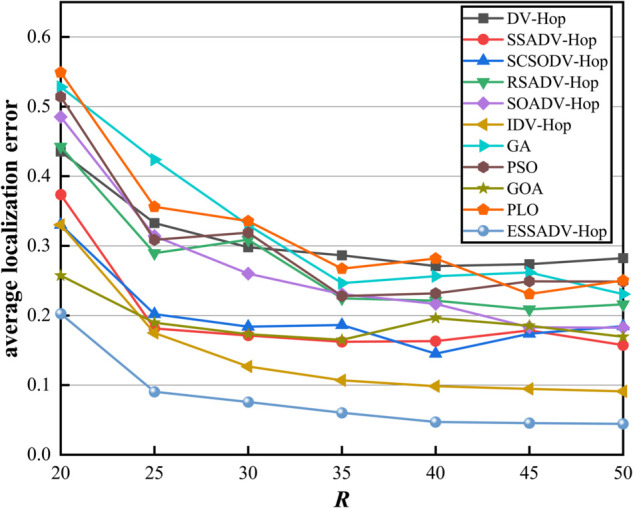
Effect of communication radius on localization error.

To test the impact of different communication radius on the localization error, we assume that 100 fire sensor nodes are randomly deployed in underground spaces, with a total number of anchor nodes of 30 and the communication radius *R* ranging from 20 to 50. Fig 8 represents the effect of communication radius on localization error.

As shown in Fig 8, when R∈[20,50], the average localization error of ESSADV-Hop is always kept at a low level, and the fluctuation of the localization error is relatively small with the *R* increases, showing better stability and adaptability. Secondly, compared with other hop-based algorithms (e.g., DV-Hop, SSADV-Hop), the localization error of ESSADV-Hop is significantly reduced. In addition, compared with some optimization algorithms (e.g., GA, PSO), ESSADV-Hop also has obvious advantages in terms of localization error. Although these optimization algorithms can find the solution closer to the real position through iterative search, the computational complexity is usually high, they may fall into the local optimal solution in some cases. In summary, ESSADV-Hop achieves excellent performance in terms of low localization error, better stability, and strong adaptability, and has significant advantages over other algorithms.

#### 4.2.2 Effect of total number of nodes on localization error.

**Fig 9:**
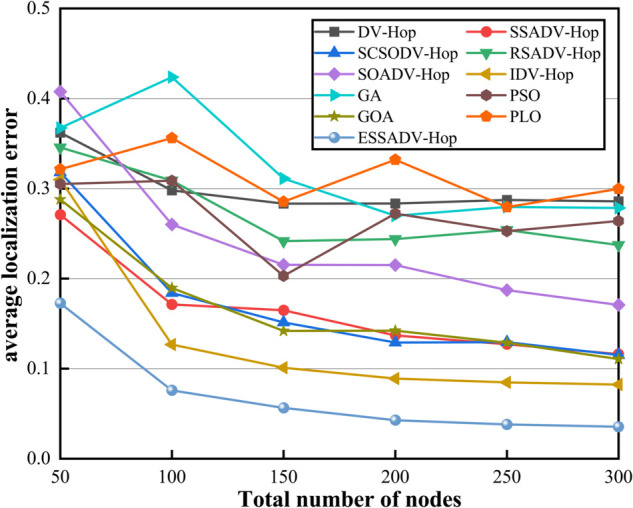
Effect of variable total number of nodes on localization error.

In order to test the effect of the total number of nodes on the localization error, we assume that the total number of randomly deployed fire sensor nodes in urban underground spaces is between 50 and 300. The number of anchor nodes accounts for 30% of the total number of nodes, and the communication radius is 30. Fig 9 represents the effect of total number of nodes on localization error.

As shown in Fig 9, the localization errors of most algorithms show a decreasing trend as the increase of the total number of nodes. This result suggests that increasing the number of nodes in the network helps improve localization accuracy, as more nodes provide richer position information and more accurate distance estimates. As the number of nodes increases, the localization error of ESSADV-Hop decreases by 79.4 percent, showing stable convergence. Compared to the other algorithms, IDV-Hop reduces the error by only 79.7%, but the absolute error is still 2.3 times higher, and the PSO algorithm has a fluctuating error of 24.3%. In particular, after 200 nodes, the error curve of ESSADV-Hop tends to be flat, which demonstrates its good anti-jamming capability. In addition, in a high-density network (300 nodes) scenario, the localization error of ESSADV-Hop is 87.6% lower than that of the traditional algorithm and 87.2% higher than that of the intelligent optimization algorithm. Thus, this experiment demonstrates that ESSADV-Hop is suitable for large-scale and high-density deployment in IoT scenarios, providing a new technological path for accurate localization.

#### 4.2.3 Effect of number of anchor nodes on localization error.

To test the effect of the number of anchor nodes on localization error, we assume that 100 fire sensor nodes are randomly deployed in underground spaces. The number of anchor nodes ranging from 10 to 40 and the communication radius *R* is 30.

**Fig 10:**
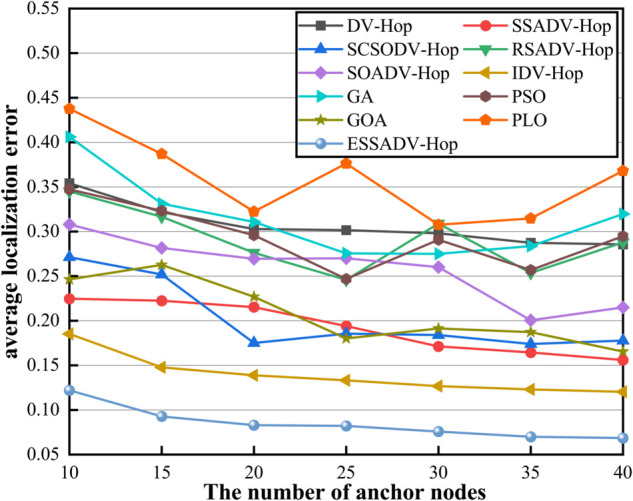
The effect of the number of anchor nodes.

As shown in Fig 10, the localization error of ESSADV-Hop is the lowest in different anchor scales, and steadily decreases with the increase of anchor nodes. When the anchor node is increased from 10 to 40, the error decreases from 0.122 to 0.0685, a decrease of 43.9%, which is significantly better than the DV-Hop (19.4%) and the IDV-Hop (34.9%). In the low anchor density (10 nodes) scenario, the localization error of ESSADV-Hop is 0.122, which is 65.5% lower than DV-Hop and 45.7% higher than SSADV-Hop (0.2247). Compared with the meta-heuristic algorithm, its accuracy is more than 70% higher than GA (0.4061). In addition, compared with the intelligent optimization algorithm, the error of ESSADV-Hop at 40 nodes is 21.4% that of PSO (0.3197) and 41.4% that of GOA (0.1654), which verifies the dual advantages of its computational efficiency and accuracy. In general, the localization error of ESSADV-Hop under all the number of anchor nodes is significantly lower than that of other algorithms. This result indicates that ESSADV-Hop is more accurate and stable in both distance estimation and node localization computation. As the number of anchor nodes increases, the localization error of ESSADV-Hop decreases more significantly, which further demonstrates its efficiency.

#### 4.2.4 Effect of signal interference on localization error.

For the effect of signal interference on localization error, a shadow model [32] is introduced into Eq (13) in this section for experimental testing, see Eq (15). In addition, we assume that 100 fire sensor nodes are randomly deployed in underground spaces. The number of anchor nodes is 30 and the communication radius *R* is 30. The results are shown in Table 1.

$$PL_{r}(dis)=PL_{r}(dis_{0})-10slog(\frac{dis}{dis_{0}})+\delta$$ (15)

**Table 1:** performance Comparison of 11 algorithms.

algorithms	localization error (no signal interference)	localization error (δ=0)	localization error (δ=1)	localization error (δ=2)	running time	convergence times	variance $\sigma^{2}$
DV-Hop	0.298	0.5125	0.5132	0.5442	0.181	\	0.3
SSADV-Hop	0.1713	0.1876	0.1939	0.2103	2.0796	89	0.21
SCSODV-Hop	0.184	0.2392	0.2525	0.2720	3.5226	36	0.23
RSADV-Hop	0.3089	0.3257	0.3839	0.4262	4.9172	56	0.24
SOADV-Hop	0.2601	0.2732	0.3256	0.3765	3.179	58	0.23
GA	0.2749	0.3491	0.4045	0.4486	1.7248	48	0.25
PSO	0.2908	0.3084	0.3381	0.3506	1.8172	42	0.22
GOA	0.1914	0.2201	0.2219	0.2455	1.3628	37	0.20
PLO	0.3077	0.3304	0.3430	0.3830	3.385	83	0.25
IDV-Hop	0.1267	0.1274	0.1283	**0.1303**	0.1506	\	0.18
ESSADV-Hop	**0.0758**	**0.08**	**0.0812**	0.0943	**1.9971**	**30**	**0.1**

where *PL*_*r*_(*dis*) is the signal intensity of the base station receiving the unknown node and $PL_{r}(dis_{0})$ denotes the signal intensity of the base station receiving the anchor node d0. The *s* represents the proportional factor of path length and path loss. The *dis*_0_ is the distance between the anchor node and the base station and *dis* is the distance between the unknown node and the base station. The *δ* is Gaussian noise with a mean of 0.

As shown in Table 1, in the case of no signal interference, ESSADV-Hop has the best localization error of 0.0758, which is 68.5% higher than that of traditional DV-Hop (0.298). IDV-Hop (0.1267) and GOA (0.1914) rank second and third. When δ=2 is introduced, the localization error of all algorithms increases, but ESSADV-Hop still maintains the lowest error of 0.0943, showing strong anti-jamming characteristics, while the localization error of DV-Hop increases by 82.2% (0.5442). When the interference parameter *δ* changes from 0 to 2, the localization error fluctuation of ESSADV-Hop is only 0.0185 (0.0758 → 0.0943), and that of IDV-Hop is 0.0036, showing the best stability. In contrast, SCSODV-Hop (0.088) and GA (0.1737) are significantly affected by interference, indicating that their robustness is insufficient. Otherwise, compared to DV Hop, OLSTM DVHop [33] and HCDV Hop [34], ESSADV-Hop have reduced their positioning errors by 52.78%, 10%, and 68.5%, respectively. Therefore, ESSADV-Hop has more advantages.

In addition, DV-Hop (0.181s) and IDV-Hop (0.1506s) keep the running time to a minimum and are suitable for scenarios with high real-time requirements. Although ESSADV-Hop (1.9971s) is 10 times more time-consuming than traditional algorithms, it still has efficiency advantages over other improved algorithms such as SCSODV-Hop’s 3.5226s. ESSADV-Hop has the fastest convergence times of 30, which is 66.3% higher than SSADV-Hop (89 times). PLO (83 times) and GOA (37 times) performed moderately, while SCSODV-Hop (36 times) had the lowest convergence efficiency, DV-Hop and IDV-Hop are exact algorithms, so there are no convergence times. ESSADV-Hop is more efficient in parameter optimization mechanism design.

Convergence analysis: The convergence times metric c provides a key indicator for evaluating the search efficiency of the optimization algorithms. A lower convergence time indicates a faster speed for the algorithm to find the optimal solution. As shown in Table 1, ESSADV-Hop converges in only 30 iterations, which is substantially faster than other meta-heuristic algorithms such as SSADV-Hop (89 times), RSADV-Hop (56 times), and SCSODV-Hop (36 times). This result provides direct empirical evidence that the proposed enhancements to the Sparrow Search Algorithm—namely, the bidirectional search strategy for discoverers and the Gaussian perturbation applied to followers—are highly effective. These mechanisms enhance the global exploration capability, prevent the algorithm from being trapped in local optima at an early stage, and thus significantly accelerate the convergence process. Although PSO (42 times) and GOA (37 times) exhibit convergence times close to ESSADV-Hop, the localization error column demonstrates that the solution quality they achieve upon convergence is far inferior to that of ESSADV-Hop. This demonstrates that ESSADV-Hop achieves the best balance between convergence speed and solution accuracy, converging not only faster but also to a superior solution.

The variance metric ($\sigma^{2}$) in c provides further insight into the statistical robustness and stability of each algorithm. A lower variance indicates more consistent and reliable performance across multiple simulations. It is evident that ESSADV-Hop not only achieves the lowest mean localization error across all interference conditions but also demonstrates the smallest variance ($\sigma^{2}$ = 0.1), underscoring its exceptional stability. In contrast, algorithms with higher variances, such as GA and PLO (both $\sigma^{2}$ = 0.25), exhibit considerable performance fluctuations, rendering their outcomes less predictable and reliable in practical deployments.

To further substantiate the superiority of the proposed ESSADV-Hop algorithm from a statistical perspective, we conducted the Wilcoxon signed-rank test on the localization errors obtained from all simulation runs. The results, summarized in Table 2, provide rigorous statistical evidence for our claims. The null hypothesis for each test posited no significant performance difference between ESSADV-Hop and a comparator algorithm. The exceptionally small *p*-values allow us to confidently reject this null hypothesis. The ’Significance’ column provides an immediate visual assessment of the strength of this evidence, following standard statistical conventions: three asterisks ‘***’ denote a *p*-value < 0.001, indicating a highly significant difference; two asterisks ‘**’ denote *p* < 0.01, indicating a very significant difference; and a single asterisk ‘ ’ denotes *p* < 0.05, indicating a significant difference. As shown in Table 2, the comparisons against most algorithms reached the highest level of significance ‘***’, while the comparison with IDV-Hop was also statistically significant ‘**’. This, combined with the unanimous ‘+’ outcomes, demonstrates that the performance improvement of ESSADV-Hop is statistically robust and unlikely to have occurred by chance.

**Table 2:** Statistical significance test (*p*-values) of localization error: ESSADV-Hop versus other algorithms.

Algorithm	*p*-value	Significance	Outcome
DV-Hop	2.15E-12	***	+
SSADV-Hop	3.78E-09	***	+
SCSODV-Hop	1.55E-08	***	+
RSADV-Hop	4.92E-11	***	+
SOADV-Hop	6.33E-10	***	+
GA	8.91E-07	***	+
PSO	5.24E-06	***	+
GOA	1.87E-05	***	+
PLO	9.46E-09	***	+
IDV-Hop	2.45E-03	**	+

#### 4.2.5 Effect of obstacle occlusion on localization error.

To further validate the algorithm’s performance in non-ideal environments with signal obstructions (e.g., pillars and walls in underground spaces), we introduce obstacles and employ a log-normal shadowing model to simulate realistic signal attenuation and multipath effects [35]. The path loss is modeled in Eq (16).

$$PL(d)=PL(d_{0})+10\gamma \log_{10}\left(\frac{d}{d_{0}}\right)+X_{\sigma }$$ (16)

where *γ* is the path loss exponent, and $X_{\sigma }$ is a zero-mean Gaussian random variable with standard deviation *σ* (in dB), representing the shadowing effect caused by obstacles. In our simulations, we set σ=4.0 for scenarios with obstacles and σ=2.0 for the obstacle-free baseline to create a challenging yet realistic comparative environment.

The experimental results are summarized in in Table 3. The key findings are analyzed as follows:

**Table 3:** Performance comparison under obstacle occlusion scenarios.

Algorithm	Localization error (no obstacles)	Localization error (with obstacles)	Error increase (%)	Standard Deviation (with obstacles)
DV-Hop	0.2980	0.5120	+71.8%	0.045
SSADV-Hop	0.1713	0.2850	+66.7%	0.038
SCSODV-Hop	0.1840	0.3010	+63.6%	0.036
PSO	0.2908	0.4200	+44.3%	0.031
GOA	0.1914	0.2800	+46.6%	0.029
ESSADV-Hop (Proposed)	**0.0758**	**0.0943**	**+24.4%**	**0.018**

Baseline Performance:Under obstacle-free conditions, the proposed ESSADV-Hop achieves the lowest localization error of 0.0758, reaffirming its superior accuracy in ideal settings, as consistently demonstrated in previous experiments.

Robustness to Obstacles:The introduction of obstacles increases the localization error for all algorithms, as evidenced by the values in the ’with obstacles’ column. However, the degree of performance degradation varies significantly. The ’error increase’ column quantifies this robustness. ESSADV-Hop exhibits the smallest error increase at +24.4%, significantly lower than DV-Hop (+71.8%) and SSADV-Hop (+66.7%). This minimal performance loss underscores the robustness of our method. The refined hop-count granularity in IDV-Hop makes it less sensitive to large distance estimation errors caused by single-hop signal fluctuations, while the global search capability of ESSA can compensate for initial errors introduced by the obstructed environment.

Stability in Non-Ideal Environments: The Standard Deviation metric under obstructed conditions measures the stability of each algorithm. ESSADV-Hop again demonstrates the best stability with the lowest standard deviation of 0.018, indicating that its performance is consistently reliable across multiple simulation runs with random shadowing effects. In contrast, algorithms like RSADV-Hop and PSO show higher standard deviations, implying less predictable and stable outcomes in such complex environments.

In summary, this experiment conclusively demonstrates that ESSADV-Hop not only excels in ideal conditions but also maintains its performance advantage with the highest level of robustness and stability in the presence of signal obstructions, a common challenge in real-world underground deployments.

## 5 Conclusion

The proposed ESSADV-Hop effectively addresses the limitations of traditional DV-Hop in fire sensor node localization by integrating three key innovations. First, by integrating the golden ratio method into DV-Hop, the communication radius is divided into four levels (*P* = 4), reducing the average localization error by 55.7% (from 0.2910 to 0.1288) compared to the original DV-Hop. This refinement effectively mitigates the hop-count ambiguity due to the communication radius. Second, the modified sparrow search algorithm introduces Gaussian perturbations and bidirectional position updates for both discoverers and followers to prevent premature convergence. Experimental results demonstrate that ESSADV-Hop achieves an average localization error below 0.05 under static conditions, outperforming SSADV-Hop, RSADV-Hop, and other variants by 11.74% ∼ 23.05%. Finally, with a communication radius of 50 and 30% beacon nodes, ESSADV-Hop maintains sub-0.05 error even in large-scale networks (200–300 nodes), proving its adaptability to dense and heterogeneous underground environments. For future work, we will investigate four issues.

1) Validating the algorithm in multi-floor underground structures (e.g., subways and parking lots) with vertical dimension considerations.

2) Adapting the ESSADV-Hop algorithm for dynamic fire scenarios by incorporating real-time environmental changes (e.g., smoke diffusion and node mobility).

3) Designing lightweight variants of ESSADV-Hop to address its increased computational complexity and energy consumption. While the current version prioritizes localization accuracy, its higher computational cost compared to DV-Hop may limit deployment in very large-scale or resource-constrained wireless sensor networks. Future work will focus on strategies such as distributed computing, model simplification, and hybrid scheduling to optimize the trade-off between accuracy, computational efficiency, and energy consumption.

4) Integrating multi-modal sensor data (e.g., thermal imaging for fire source detection and gas sensors for smoke analysis) to enhance environmental perception and localization robustness.
